# CryoTriplane: triplane-based neural radiance field representation for high-resolution cryo-EM 3D homogeneous reconstruction

**DOI:** 10.1093/bioinformatics/btag754

**Published:** 2026-10-13

**Authors:** Yiran Cheng, Bintao He, Qiong Zeng, Fa Zhang, Renmin Han

**Affiliations:** Frontiers Science Center for Nonlinear Expectations (Ministry of Education), Research Center for Mathematics and Interdisciplinary Sciences, Qilu Hospital (Qingdao), Cheeloo College of Medicine, Shandong University, Qingdao 266237, China; College of Medical Information and Engineering, Ningxia Medical University, Yinchuan 750004, China; Frontiers Science Center for Nonlinear Expectations (Ministry of Education), Research Center for Mathematics and Interdisciplinary Sciences, Qilu Hospital (Qingdao), Cheeloo College of Medicine, Shandong University, Qingdao 266237, China; School of Computer Science and Technology, Shandong University, Qingdao 266237, China; School of Medical Technology, Beijing Institute of Technology, Beijing 100190, China; Frontiers Science Center for Nonlinear Expectations (Ministry of Education), Research Center for Mathematics and Interdisciplinary Sciences, Qilu Hospital (Qingdao), Cheeloo College of Medicine, Shandong University, Qingdao 266237, China; College of Medical Information and Engineering, Ningxia Medical University, Yinchuan 750004, China

## Abstract

**Motivation:**

Determining the three-dimensional (3D) structure of proteins is a cornerstone of structural biology, providing essential insights into their underlying biological mechanisms and functional roles. Cryogenic electron microscopy (cryo-EM) single-particle analysis allows researchers to investigate biomolecules at near-atomic resolutions, unraveling the structure of biomolecular complexes. However, reconstructing high-fidelity 3D volumes from extremely noisy projections, particularly under the interference of structural heterogeneity, remains a challenging task. Traditional Fourier-domain methods often suffer from frequency-dependent signal attenuation and uneven information distribution. While recent deep-learning-based frameworks attempt to predict high-frequency components directly in frequency space, they are highly susceptible to noise and often introduce artifacts. State-of-the-art (SOTA) real-space method circumvents Fourier-domain defects, yet remains constrained by the inherent spectral bias of multi-layer perceptrons, as fixed positional encoding fails to sufficiently capture intricate high-frequency details.

**Results:**

We propose cryoTriplane, a real-space 3D homogeneous reconstruction network that utilizes a hybrid spatial representation to adaptively capture fine structural details. By combining fixed positional encoding for noise-robust structural initialization with structure-aware positional encoding for high-frequency refinement, our method achieves 3D reconstructions that are simultaneously globally consistent and locally fine-grained. Comprehensive qualitative and quantitative evaluations across five simulated and four real-world datasets demonstrate that cryoTriplane significantly outperforms existing methods in resolving fine structural features. Furthermore, validation through downstream atomic modeling underscores cryoTriplane’s ability to recover high-quality density maps. Specifically, our method achieves sequence coverage improvements of 4.6% and 5.5% on the EMPIAR-10005 and EMPIAR-10492 datasets over the SOTA approach, respectively.

**Availability and implementation:**

Our code is available at https://github.com/chengyr1999/CryoTriplane. The real datasets used in this study are publicly available from EMPIAR.

## 1 Introduction

Cryogenic electron microscopy (Cryo-EM) has emerged as a pivotal technique for determining the three-dimensional (3D) structures of biological macromolecules, providing electron density maps that serve as the direct basis for atomic-level structural modeling. In the case of intermediate resolution cryo-EM maps, automatic model building tools such as EMBuild (He *et al.* 2022) and DiffModeler (Wang *et al.* 2024) fit the monomeric structures predicted by AlphaFold2 (Jumper *et al.* 2021) to the backbone structure extracted from the density map. However, for higher-resolution maps, tools like ModelAngelo (Jamali *et al.* 2024) and cryoREAD (Wang *et al.* 2023) can perform automated *de novo* modeling based only on sequence information. According to ModelAngelo, regions with a local resolution worse than 3.5–4.0 Å require manual inspection to ensure the reliability of the atomic model. This implies that fully automated atomic modeling is fundamentally dependent on achieving high-resolution cryo-EM density maps both globally and locally. To reach this requisite resolution, reconstruction methods must accurately interpret 3D information captured in two-dimensional (2D) projection images, which traditionally rely on the theoretical foundation of the central slice theorem.

As the theoretical cornerstone of single-particle analysis (SPA), the central slice theorem (Crowther *et al.* 1970) states that the Fourier transform of a 2D projection taken at a specific orientation is equivalent to a central section through the 3D Fourier transform of the object, perpendicular to the direction of projection. Based on this principle, traditional methods such as cryoSPARC (Punjani *et al.* 2017) and RELION (Scheres 2012) reconstruct 3D structures by integrating a large number of particle images in Fourier space. This process gradually fills the 3D Fourier volume, which is subsequently converted back into real space via an inverse Fourier transform. However, significant discrepancies exist between the experimental data and this theoretical ideal. Although the random orientation of particles in ice can approximate a uniform distribution to some extent, the actual acquired projection angles are inherently finite and discretely sampled. This results in an irregularly dense sampling at low frequencies and sparse coverage at high frequencies in Fourier space, as shown in Figure 1. Moreover, interpolating this irregularly sampled data onto Cartesian grid during reconstruction introduces numerical errors, thereby degrading the high-frequency resolution (Magnusson *et al.* 1992).

**Figure 1:**
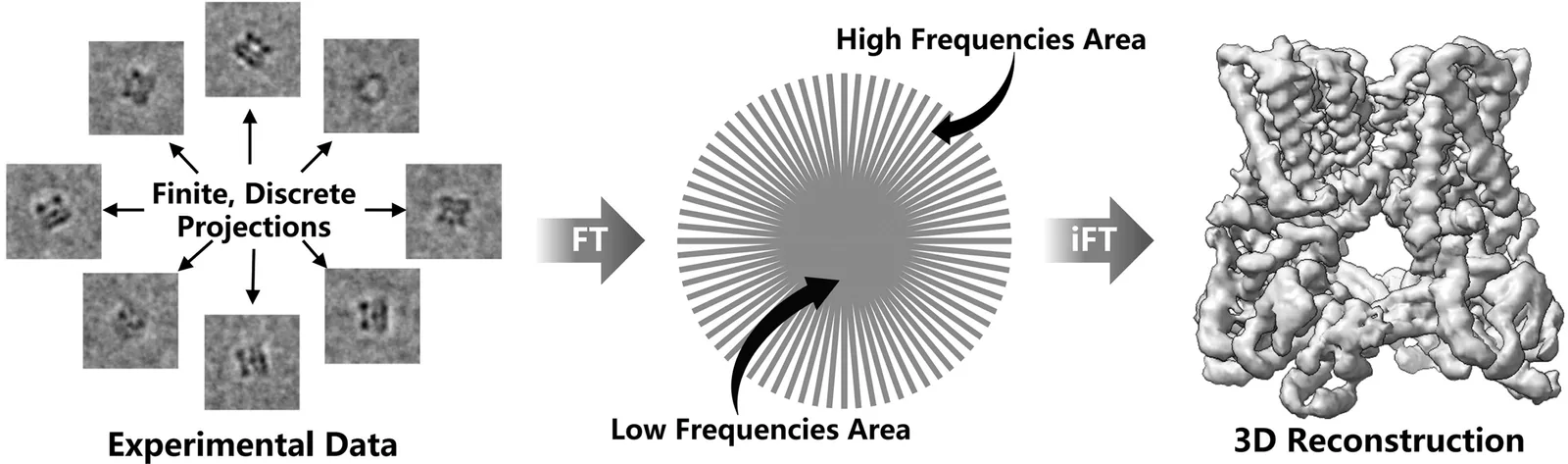
Principle of 3D volume reconstruction in Fourier space.

To overcome the inherent limitations of discrete grid-based interpolation, there exists a growing need for a continuous paradigm for 3D volumetric representation. In this context, the breakthrough of implicit neural representations in computer vision, particularly the success of neural radiance field (NeRF), has demonstrated its capability to model continuous scenes without relying on discrete grids. This provides a reference framework for applying deep learning methods to cryo-EM 3D reconstruction. With known particle poses and contrast transfer function (CTF) parameters as inputs, cryoDRGN (Zhong *et al.* 2021) was the first to adapt a NeRF-like framework for homogeneous cryo-EM reconstruction and to additionally incorporate the variational autoencoder for resolving structural heterogeneity. The enhancement of high-frequency spatial coordinate representation via positional encoding, coupled with the implicit modeling of the 2D Fourier slice by multi-layer perceptrons (MLPs), enables this model to directly estimate density information in different frequency spatial coordinates. However, cryoDRGN still relies on the Fourier-domain reconstruction paradigm, making it difficult to overcome the problem of irregular information distribution in Fourier space. To address this limitation, cryoNeFEN (Huang *et al.* 2024) transforms the representation of the neural field from the Fourier space into the real space. This method directly optimizes the 3D density volume through differentiable rendering, formulating the projection operation as an integral of density along ray directions. Therefore, it avoids the dependence on Fourier spatial uniformity and achieves isotropic, high-resolution reconstruction. This method significantly improves the global and local resolution of reconstructed density maps, offering a reliable foundation for downstream structural modeling.

Motivated by the goal of high-resolution reconstruction, we analyze the applicability of the NeRF architecture in real-space cryo-EM 3D reconstruction, with a specific focus on its capacity to represent complex high-frequency structural details. According to the neural tangent kernel theory, neural networks optimized in the real domain exhibit an inherent spectral bias, whereby they tend to learn low-frequency signals. The limitation of using only 3D coordinate vectors as MLPs input is that the smooth inductive bias of the model produces similar density estimates for adjacent coordinates (Rahaman *et al.* 2019). These results in an overly smooth density distribution that fails to recover high-frequency details and ultimately exhibits a low-frequency-dominated signal reconstruction. To address this issue, NeRF introduces the sinusoidal positional encoding mechanism, which maps the original spatial coordinates into a high-dimensional Fourier feature space. This encoding strategy significantly expands the differences between adjacent sampling points in the representation space, thereby improving the sensitivity and reconstruction ability of the model to higher-frequency spatial changes. Nevertheless, the recovery of such signals remains challenging in cryo-EM due to the low signal-to-noise ratio (SNR) of the projection images, which further makes it difficult to capture authentic high-frequency information in real space.

Here, we propose a cryo-EM 3D homogeneous reconstruction network called cryoTriplane that introduces a hybrid adaptive encoding mechanism into the coordinate mapping process. Building upon the traditional sinusoidal encoding, cryoTriplane incorporates a learnable triplane-based feature representation, which is iteratively refined through gradient descent. This mechanism effectively implements an adaptive position encoding driven by structural features, in contrast to the global, fixed-frequency mapping used across the entire spatial domain. This fusion of real-space structural consistency and adaptive detail capture allows cryoTriplane to achieve substantially superior results on simulated and real-world datasets, evidenced by significant gains in both Gold-Standard (GS) and map-to-map FSC metrics. Importantly, this higher-quality reconstruction provides direct benefits for biological analysis, which is confirmed in the *de novo* modeling experiments on the real-world datasets. In summary, our main contributions are as follows:

- We propose a novel high-resolution cryo-EM 3D homogeneous reconstruction network called cryoTriplane, aiming at reconstructing high-frequency detail information from low SNR 2D projection images.
- We propose a structure-aware positional encoding strategy that integrates triplane representations with sinusoidal encodings to map spatial coordinates into a high-dimensional feature space, adaptively reflecting high-frequency structural information.
- We visually and quantitatively evaluate cryoTriplane on both simulated and real-world datasets to demonstrate its superiority in high-resolution 3D reconstruction and its effectiveness in facilitating subsequent *de novo* modeling.

## 2 Method

### 2.1 Cryo-EM 3D reconstruction model

We employ a continuous neural field representation to model the 3D cryo-EM density map. Unlike discrete voxel grids, this approach parameterizes the underlying density as a continuous function defined on $R^{3}$. For cryo-EM SPA datasets, each 2D experimental projection $I_{i}$ is associated with a known spatial pose derived from the homogeneous refinement step. To calculate the volumetric density from the perspective of the *i*-th particle image, canonical spatial coordinate *X* needs to be transformed into the corresponding position via a rigid transformation parameterized by the known particle rotation $R_{i}\in SO(3)$ and translation $T_{i}\in R^{2}$. Then, positional encoding PE(·) is employed to embed the input coordinates into a higher-dimensional feature space, allowing the network to represent intricate, high-frequency details of the scene. Finally, these feature vectors are decoded by MLPs F(·), thus mapping the features to the volumetric density for the corresponding 3D coordinate. Consequently, the complete volumetric density field for pose $\left(R_{i},T_{i}\right)$, denoted by $V_{i}$, is formulated by aggregating the individual point-wise predictions as follows:


(1) $$V_{i}=F(\text{PE}(R_{i}X+T_{i})).$$

### 2.2 Structure-aware positional encoding

The introduction of fixed feature mapping, particularly Fourier features, marked a significant breakthrough in enabling MLPs to represent high-frequency signals, owing to its simplicity and high computational efficiency. Inspired by this, cryoDRGN also employs a fixed encoding scheme. It utilizes a combination of *L* sinusoidal curves with wavelengths geometrically progressing from 1 to the Nyquist limit for position encoding, providing the model with a set of frequency basis functions pe(·) that match the physical properties of cryo-EM particle data in the Fourier domain. This scheme can be formulated as:


(2) $${\text{pe}}^{\left(2i\right)}(x)=\text{sin}\;\left(D\pi {\left(\frac{2}{D}\right)}^{\frac{i}{L-1}}x\right),i=0,1,\ldots ,L-1,$$

(3) $${\text{pe}}^{\left(2i+1\right)}(x)=\text{cos}\;\left(D\pi {\left(\frac{2}{D}\right)}^{\frac{i}{L-1}}x\right),i=0,1,\ldots ,L-1.$$

where x is the normalized coordinates normalized and *D* is the image size.

To further enhance the high-resolution structural representation capability of the model, cryoTriplane innovatively proposes a hybrid, structure-aware representation strategy that integrates an adaptive triplane-based feature grid with the fixed positional encoding, as shown in Fig. 2. This design achieves the complementary representation of low- and high-frequency information. On the one hand, it leverages fixed positional encoding to establish a stable and reliable foundation for low-frequency reconstruction, ensuring the robustness of structure estimation even under severe low SNR conditions. On the other hand, it incorporates the adaptive triplane representation as a dynamic supplement for high-frequency features, enabling the model to extract complex, high-resolution details in regions with sufficient signal.

**Figure 2:**
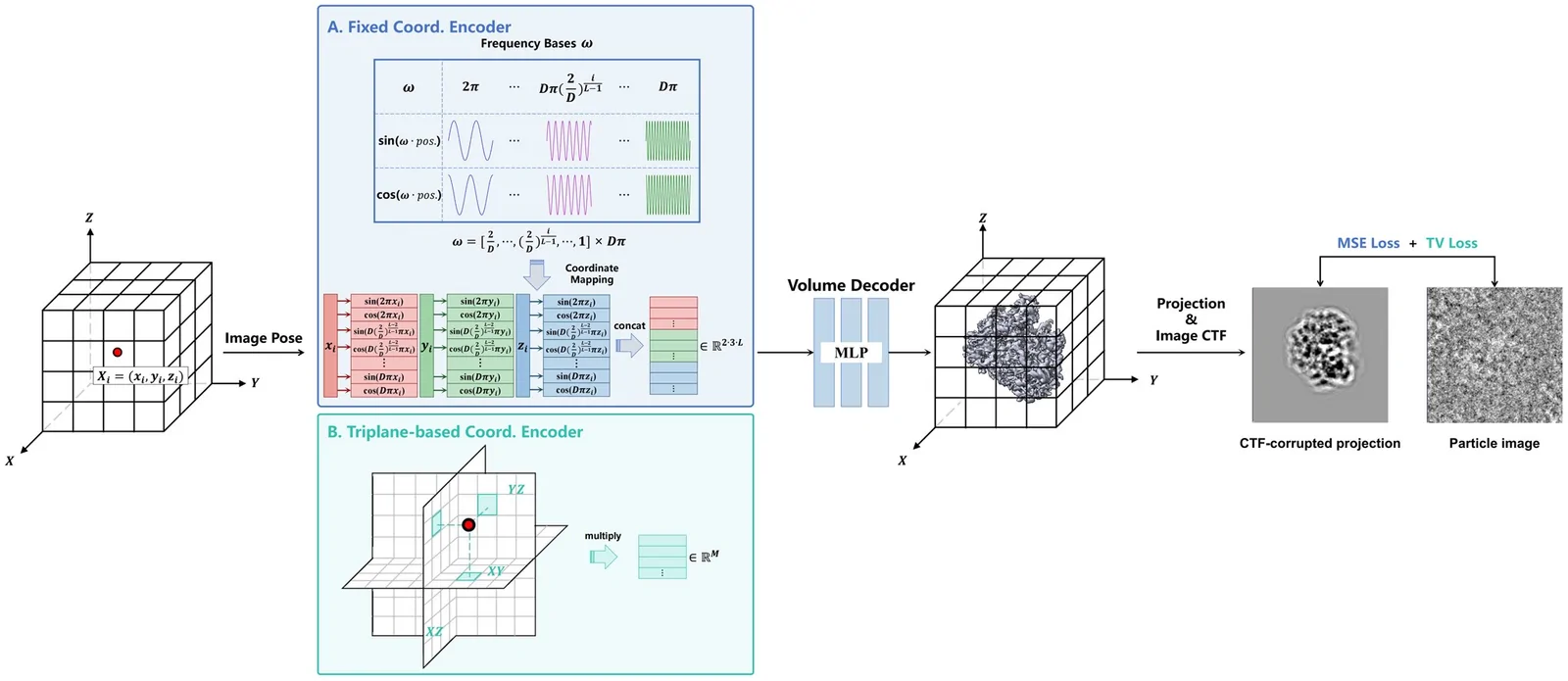
Overview of cryoTriplane. CryoTriplane combines a structure-aware positional encoding strategy with a real-space NeRF model to reconstruct a high-resolution 3D cryo-EM volume from 2D particle images. Inspired by the imaging physics model, the standardized 3D coordinates are first transformed according to the pre-estimated particle pose. The transformed coordinates are then encoded by (A) Fixed Coord. Encoder and (B) Triplane-based Coord. Encoder, whose outputs are concatenated to form a hybrid adaptive representation. This representation is subsequently decoded by MLPs to generate a 3D density map. The predicted density map is then projected into 2D image space and modulated by the CTF parameter. Finally, the model is optimized by minimizing the MSE loss between the observed particle images and the generated projections, together with a TV regularization loss.

Specifically, the triplane-based representation, denoted as *T*, is composed of three learnable, orthogonal feature planes $\left(T_{xy},T_{yz},T_{xz}\right)$. Each feature plane has dimensions of D×D×t, where each grid point stores a feature vector of dimension *t* (set to 16). These planes serve as a spatially explicit memory for capturing high-frequency details that supplement the global structure. To query the feature vector $w\in R^{t}$ at a given 3D coordinate (x,y,z), the coordinate is first projected onto each of the three planes, followed by bilinear interpolation to yield features $\left(w_{xy},w_{yz},w_{xz}\right)$, which are then aggregated via element-wise multiplication $w=w_{xy}\times w_{yz}\times w_{xz}$, inspired by the feature fusion mechanism in K-planes (Fridovich-Keil *et al.* 2023). This multiplicative aggregation enforces a joint spatial constraint by confining activations to the intersection of all three planes, thereby directly modeling multi-dimensional spatial correlations and capturing compact, high-frequency structures. The final hybrid representation PE(x,y,z) is formulated by concatenating the triplane-based feature with the fixed positional encoding: PE(x,y,z)=[pe(x,y,z),w].

### 2.3 Training procedure

The forward projection model describes the physical process by which a 3D density map, *V*, is projected to form a 2D particle projection in cryo-EM (Bendory *et al.* 2020). This generative process can be divided into the following steps. First, the 3D volume is oriented according to the sampled rotation matrix *R*. A clean 2D projection is then generated by integrating the rotated volume along the projection axis (conventionally the *z*-axis). Subsequently, this projection is shifted within its plane by a translation operator *T*. To account for the microscope’s optical aberrations, the shifted projection is then convolved with the CTF parameters *C*. Finally, additive Gaussian noise, *n*, is introduced to simulate the low-SNR conditions, yielding the final projection image *I*. This entire process can be mathematically expressed as:


(4) $$I=C*T_{i}\int_{-\infty }^{+\infty }RV+n,$$

where * denotes the convolution operation.

For computational implementation, we first generate a clean 2D projection for the *i*-th particle which is achieved by applying its corresponding 3D rotation $R_{i}$ to the volume $V_{i}$ and then discretely summing the rotated voxel values along the projection axis. The resulting clean projection is then transformed into Fourier space to be multiplied with the corresponding CTF $C_{i}$. The generation of predicted projection images can be formulated as (Huang *et al.* 2024):


(5) $$\hat{I_{i}}=F^{-1}(F(C_{i})F(\sum_{z=1}^{D}V_{i}(x,y,z))),$$

where F(·) and $F^{-1}(\cdot )$ represent the Fourier transform and the inverse Fourier transform, respectively.

### 2.4 Loss function

The network is trained by minimizing a total objective function, which consists of a reconstruction loss and a regularization term. The reconstruction loss $L_{\text{rec}}$ is a pixel-level mean squared error (MSE) that enforces fidelity to the experimental observations $I_{i}$. It quantifies the difference between the predicted image $\hat{I_{i}}$ and the ground truth, and is given by:


(6) $$L_{\text{rec}}(I_{i},\hat{I_{i}})=||I_{i}-\hat{I_{i}}|{|}_{2}^{2}$$

For the regularization loss $L_{\text{TV}}$, it aims to prevent the model from overfitting to the high-frequency noise inherent in the experimental data. This term encourages spatial smoothness in the predicted image ${\hat{I}}_{i}$ while preserving sharp edges. We employ the total variation loss to impose constraints on the model’s fitting capacity, which can be formulated as:


(7) $$L_{\text{TV}}({\hat{I}}_{i})=\sum_{x,y}(({\hat{I}}_{i}(x+1,y)-{\hat{I}}_{i}(x,y){)}^{2}+$$

(8) $${\left({\hat{I}}_{i}(x,y+1)-{\hat{I}}_{i}(x,y)\right)}^{2}{)}^{1/2}.$$

Thus, the total optimization objective can be formulated as:


(9) $$L(I_{i},\hat{I_{i}})=L_{\text{rec}}(I_{i},\hat{I_{i}})+\alpha L_{\text{TV}}(\hat{I_{i}}).$$

where α is a constant weight, set to $\alpha =10^{-3}$.

## 3 Results

### 3.1 Datasets

#### 3.1.1 Simulated datasets

To comprehensively evaluate the reconstruction performance of cryoTriplane, we generate five synthetic datasets that closely reflect the complexities of real-world cryo-EM data. The data generation process begins with a 3D density map, which is generated based on the heterodimeric ABC exporter TmrAB atomic model (PDB: 6RAK) using the molmap command in UCSF ChimeraX (Pettersen *et al.* 2004). To ensure that the simulated projection angles reflect realistic experimental distributions, pose and CTF parameters are extracted from the EMPIAR-10049.star file. These parameters are subsequently employed to generate 5000 projection images across diverse viewing angles within cryoSPARC.

Next, three typical real-world challenges are introduced into the dataset construction:

- Noise Level: Cryo-EM images inherently suffer from extremely low SNR to minimize radiation damage to specimens. To simulate these challenging real-world conditions, the default SNR for our simulated datasets was set to 0.05, and further reduced to 0.01 for designated low-SNR experiments.
- Pose Inaccuracy: High-resolution cryo-EM reconstruction demands precise particle poses, but existing estimation methods are often imperfect due to image noise. Accumulated rotational and translational errors ultimately degrade resolution and blur high-frequency details. To evaluate model robustness against this inherent noise, random rotational errors ranging from −1° to 1° were applied to each Euler angle, alongside random spatial shifts of ±1 pixel along the *X* and *Y* axes.
- Conformational Heterogeneity: Cryo-EM samples frequently contain mixtures of conformational states, which blur reconstructions if treated uniformly. To simulate this heterogeneity, structures corresponding to distinct states (PDB IDs: 6RAH, 6RAI, 6RAJ, and 6RAL) are combined to generate a realistic mixed projection dataset at a ratio of 2:3:2:3.

Finally, to evaluate the reconstruction quality under the above challenges, five simulated datasets are constructed, and the comprehensive workflow for generating these datasets is illustrated in Fig. 3.

**Figure 3:**
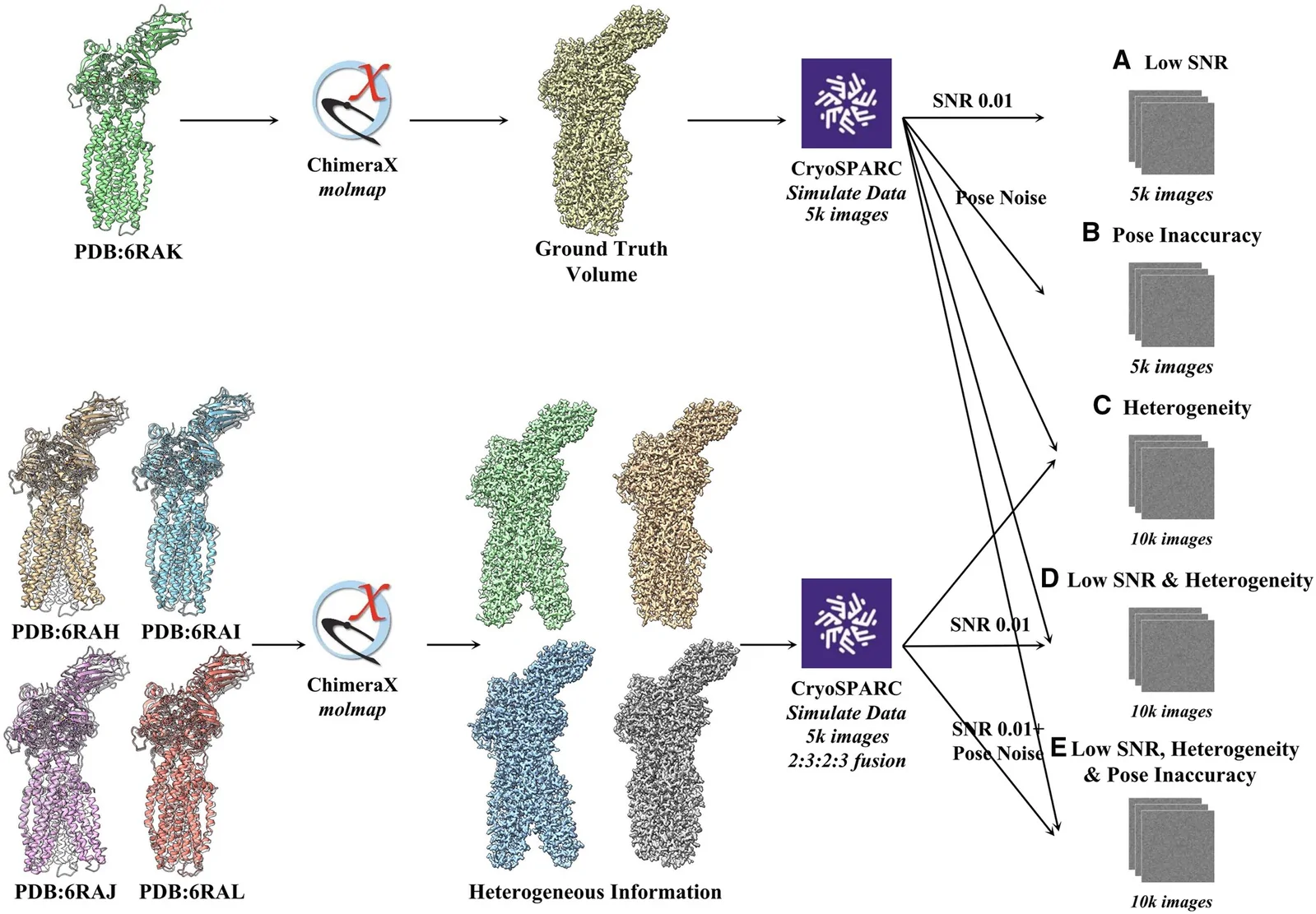
Overview of simulated datasets. Five simulated datasets were designed to evaluate the performance of cryoTriplane under various challenging conditions: (A) low SNR, (B) pose inaccuracy, (C) structural heterogeneity, (D) low SNR combined with heterogeneity, and (E) low SNR with both heterogeneity and pose inaccuracy. For data generation, the ground truth volume for the target homogeneous reconstruction and quantitative evaluation is derived from PDB: 6RAK, whereas additional simulated maps from PDBs: 6RAH, 6RAI, 6RAJ, and 6RAL are utilized to introduce structural heterogeneity.

#### 3.1.2 Real-world datasets

To evaluate the performance of cryoTriplane under realistic condition, four experimental datasets from the EMPIAR database are employed to assess and compare the reconstruction capabilities of various methods, including EMPIAR-10005 (Liao *et al.* 2013), EMPIAR-10126 (Autzen *et al.* 2018), EMPIAR-10492 (Ke *et al.* 2020), and EMPIAR-10786 (Harris *et al.* 2022).

#### 3.1.3 Data processing details

Consensus reconstruction is performed within cryoSPARC for pose estimation and mask generation on real-world datasets. Specifically, we perform the “ab initio reconstruction” job with default parameters and follow this with the “homogeneous refinement” job incorporating the corresponding symmetry information to obtain precise particle orientations and a consensus mask. This consensus mask is utilized as a loose spatial bounding box for training acceleration and quantitative evaluation. Additionally, the CTF parameters are sourced directly from the EMPIAR datasets. To guarantee the strict independence of the resolution estimation, we first divide particle images into two disjoint half-datasets according to the “RandomSubset” labels from the EMPIAR metadata. Furthermore, the training of each half-map network is executed fully independently and terminates at a fixed and pre-determined number of iterations. Finally, all evaluated methods utilize identical input elements (particles, consensus poses, CTF parameters, and masks) and are assessed with the same B-factor value during post-processing in RELION.

### 3.2 Evaluation metrics

- GS FSC: The reconstruction resolution is assessed using the Gold-Standard Fourier Shell Correlation (GS FSC) (Rosenthal and Henderson 2003). This metric involves independently reconstructing two half-maps from a randomly partitioned dataset. The final resolution is determined at the spatial frequency where the FSC curve between these maps intersects the 0.143 criterion.
- Map-to-map FSC: The map-to-map Fourier Shell Correlation is used to evaluate the structural similarity between the final averaged reconstructed map and the ground truth volume. Typically, the spatial frequency corresponding to the FSC threshold of 0.5 is used to define the resolution of reliable structural agreement.
- Local resolution: The local resolution analysis within the RELION software (Kucukelbir *et al.* 2014) is performed to assess the resolution variability across the reconstructed density map. It computes the FSC between two independent half-maps across local regions, defining the resolution at each voxel using the 0.143 criterion to evaluate structural quality throughout the volume.
- Sequence coverage: The sequence coverage is introduced to evaluate the improvement of *de novo* modeling resulting from the quality of the reconstructed density map. It is defined as the fraction of Cα atoms in the native structure that are within a certain distance cutoff (set to 3.0 Å) to any Cα atoms in the model (Terashi and Kihara 2018).

## 4 Result

### 4.1 Comparisons on simulated datasets under different conditions

To evaluate the performance of cryoTriplane under various reconstruction prior conditions, five simulated datasets are designed for comparison. The reconstructed density maps generated by cryoDRGN, cryoNeFEN, and cryoTriplane for each condition are presented in Fig. 4. Furthermore, map-to-map FSC curves are calculated for each method against the ground truth volume and are displayed in Fig. 5, alongside the GS FSC curves.

**Figure 4:**
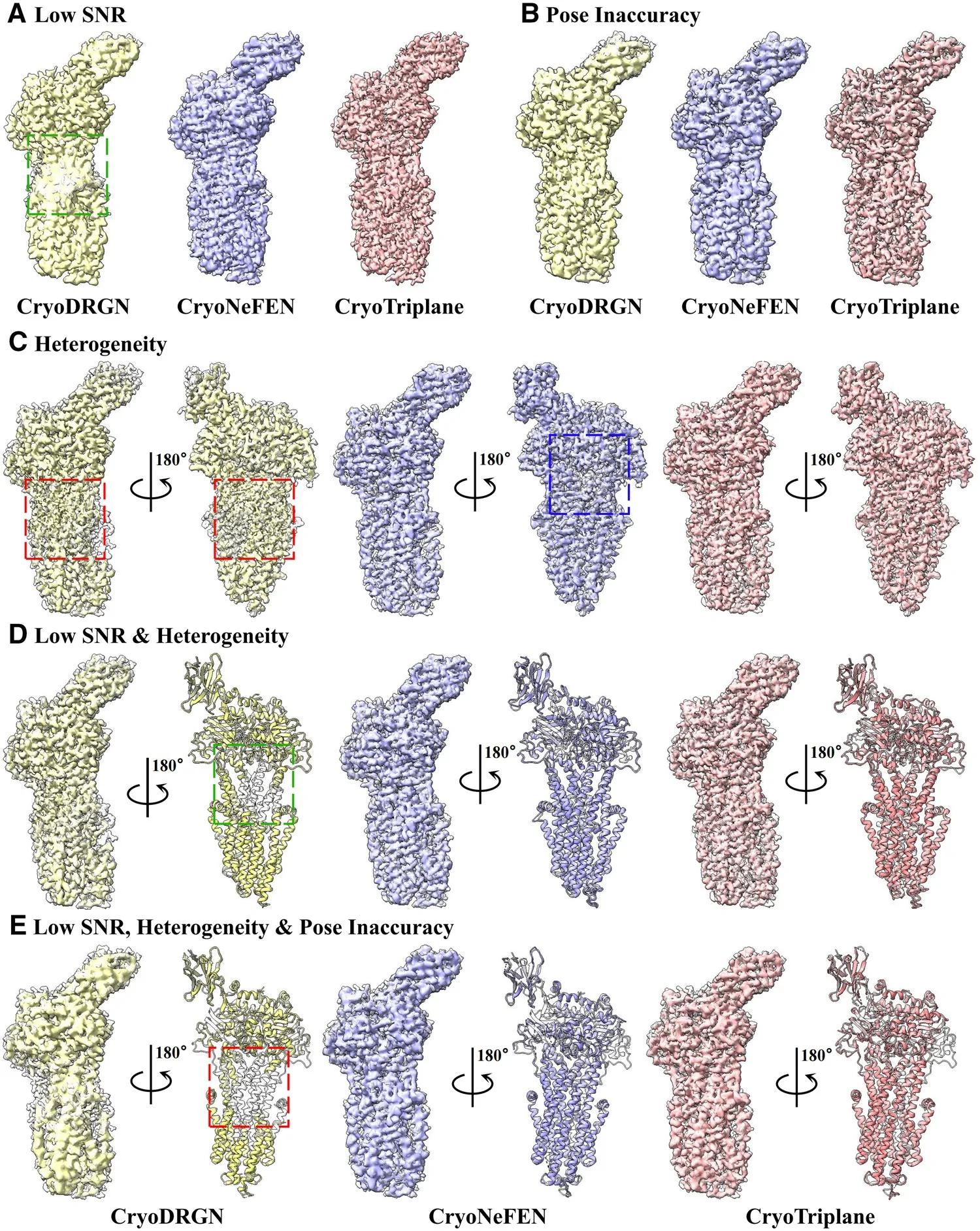
Visual comparison on simulated datasets under different conditions. Each reconstructed density map is aligned with the ground truth volume, and the derived atomic models are superimposed onto the ground truth structure (PDB: 6RAK). Dashed boxes highlight regions with structural artifacts, missing densities, or incomplete modeling results.

**Figure 5:**
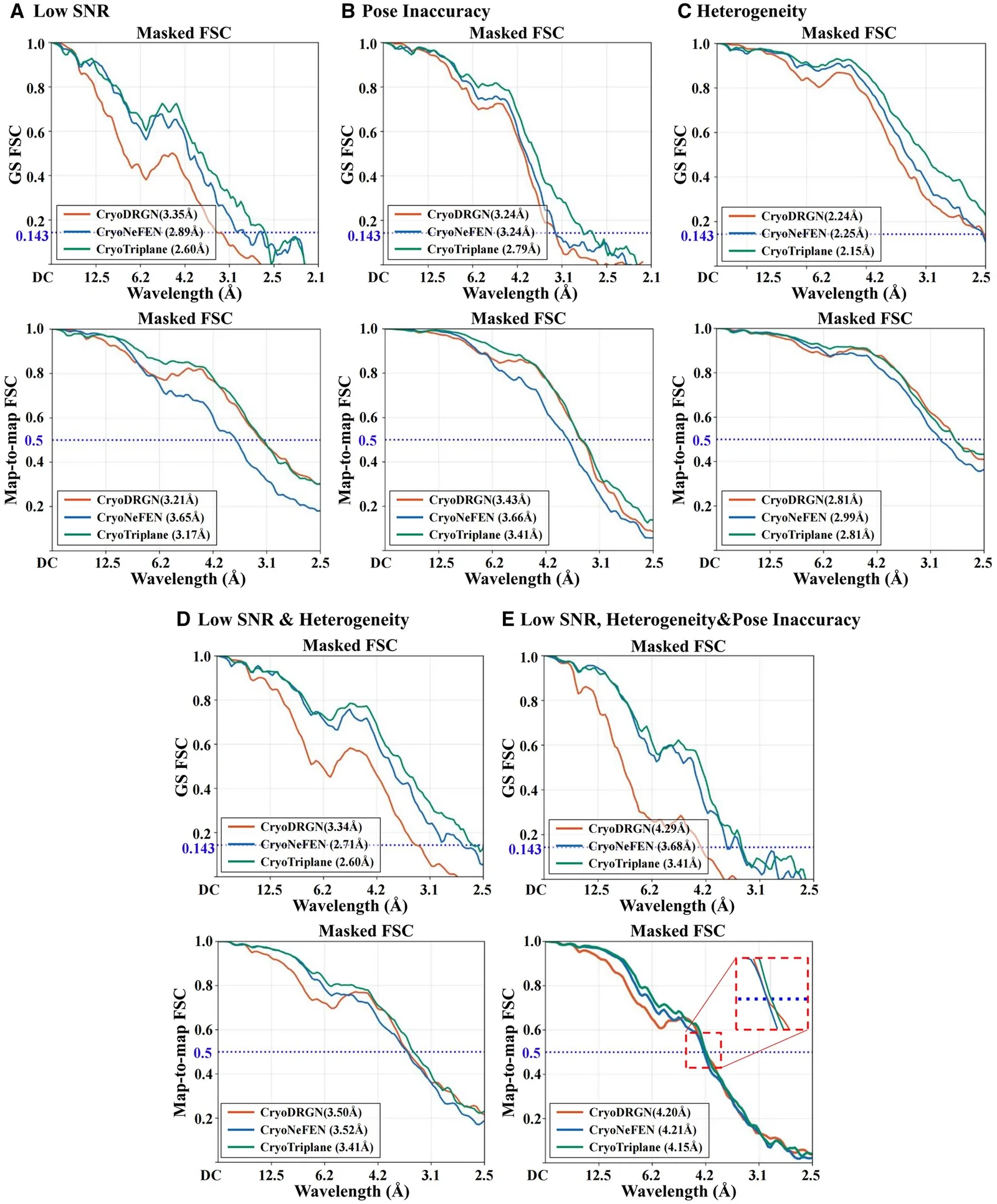
GS FSC and map-to-map FSC results on simulated datasets under different conditions. Lower GS FSC_0.143_ resolution indicates better reconstruction quality, and lower map-to-map FSC_0.5_ resolution demonstrates higher similarity to the ground truth map.

Figures 4A and 5A present a comparative analysis of visual and quantitative performance under low SNR conditions. As highlighted by the green dashed boxes in Fig. 4A, cryoDRGN produces overly smoothed density maps, revealing an inherent limitation in resolving fine structural details within high-noise backgrounds. While both real-space methods (cryoNeFEN and cryoTriplane) recover globally consistent structures, they diverge significantly in their resilience to high-frequency noise. CryoNeFEN exhibits pronounced sensitivity to severe noise interference, resulting in a relatively smooth reconstruction result and an attenuation of high-resolution details. In contrast, cryoTriplane demonstrates superior robustness, effectively recovering intricate features that are typically obscured or sacrificed in high-noise conditions. Quantitative metrics in Fig. 5A further elucidate these observations. Interestingly, cryoDRGN achieves a map-to-map FSC_0.5_ comparable to cryoTriplane, suggesting a natural proficiency in capturing the global structural envelope due to its direct modeling in Fourier space. However, this global accuracy does not translate to high-frequency consistency; the corresponding GS FSC_0.143_ is relatively lower. This discrepancy stems from the extensive noise damage to high-frequency spatial information, which ultimately leads to the observed over-smoothing after post-processing.

In scenarios involving pose inaccuracy and structural heterogeneity, cryoTriplane consistently outperforms other methods. As shown in Figs 4B and 5B, cryoTriplane obtains relatively fine reconstructed density maps and higher resolutions with the interference of only inaccurate pose. According to the red dashed box in Fig. 4C, cryoDRGN suffers from severe structural degradation and generates fragmented local densities, which subsequently compromises *de novo* atomic modeling. Similarly, cryoNeFEN fails to reconstruct valid structural information in local regions (blue box, Fig. 4C). Conversely, cryoTriplane provides highly reliable reconstructions, as evidenced in Fig. 4D and E. To assess the practical utility of these enhanced reconstructions for downstream structural analysis, we employ cryoAtom (Su *et al.* 2026) to perform atomic modeling. Notably, compared to cryoNeFEN, our method improves the sequence coverage of the resulting atomic models from 88.1% to 90.1% and from 70.0% to 75.2%, respectively.

The performance differences between cryoNeFEN and cryoTriplane are further reflected in the FSC-related comparisons, where cryoNeFEN always remains at a disadvantage. This inferior performance is primarily attributed to the insufficient representational bandwidth of fixed-encoding methods, which fail to capture high-frequency details and result in blurred reconstructions. Collectively, our proposed adaptive triplane-based strategy combines the real-space consistency with multi-frequency accuracy. By dynamically allocating representational capacity to regions with complex geometry, cryoTriplane not only achieves superior high-resolution consistency at GS FSC_0.143_ but also preserves higher frequency structural information, resulting in superior structural similarity at map-to-map FSC_0.5_. These findings collectively establish cryoTriplane as a robust framework for density reconstruction, for it demonstrates superior performance under various challenging conditions.

### 4.2 Comparisons on real-world datasets

To assess the performance of cryoTriplane in real-world scenarios, comparative results across four experimental datasets are presented below:

#### 4.2.1 EMPIAR-10005

This dataset comprises 35 645 particle images of the transient receptor potential (TRP) channel, with a box size of 256×256 pixels and a pixel size of 1.2156 Å. Figure 6A shows the reconstruction maps generated by cryoSPARC, cryoDRGN, cryoNeFEN, and cryoTriplane with C4 symmetry imposed, which are colored according to their local resolutions. To highlight structural details, black boxes are used to indicate the β-sheet region within the cytosolic domain of TRPV1, with corresponding magnified views provided below. These results reveal that cryoTriplane yields visibly sharper and more refined reconstructions, and then significantly facilitates more complete atomic modeling, as illustrated by the blue box in Fig. 6C. Figure 6B presents the global and local resolution estimates. Notably, cryoTriplane exhibits a significant improvement in resolution, achieving a 0.59 Å gain in the GS FSC_0.143_ relative to cryoNeFEN. Specifically, it reaches peak resolutions of 2.73 Å, with local resolutions ranging from 2.43 Å to 3.95 Å. As depicted in Fig. 6C, the high-fidelity density maps produced by cryoTriplane substantially improve the quality of the atomic modeling. Quantitatively, compared to cryoNeFEN, cryoTriplane increases the sequence coverage from 70.2% to 74.8%. This improvement demonstrates that the superior high-frequency details captured by cryoTriplane directly translate into more complete and accurate atomic models. Figure 6D presents the heatmaps of the channel-wise L2 norm derived from the triplane representation across three orthogonal views. These visualizations corroborate that cryoTriplane achieves a structure-aware positional encoding, as it directs richer representational capacity toward structurally dense areas.

**Figure 6:**
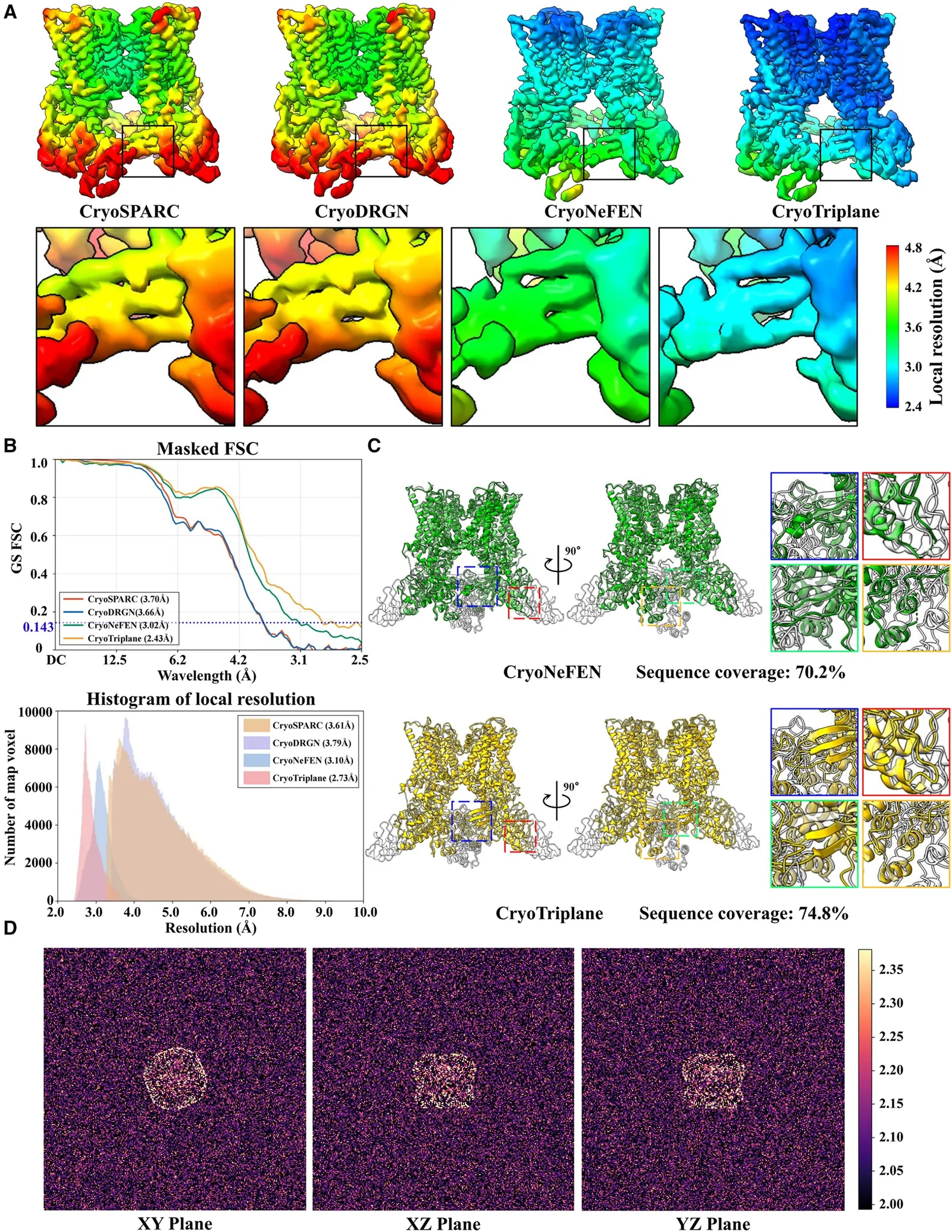
Results on TRPV1 channel (EMPIAR-10005). (A) Homogeneous reconstruction density maps generated by cryoSPARC, cryoDRGN, cryoNeFEN, and cryoTriplane under C4 symmetry. And each volume is colored according to local resolution to demonstrate the differences in reconstruction quality. The detailed view of the box area shows the reconstruction differences in the β-sheet area. (B) Plots of GS FSC curves and histograms of local resolution for maps reconstructed across different methods. (C) The atomic modeling results of cryoNeFEN and cryoTriplane under C4 symmetry, overlaid onto the reference PDB: 3J5P for comparison. The detailed views of the box region highlight the modeling difference. cryoTriplane improves the modeling sequence coverage by 4.6% compared to cryoNeFEN. (D) The heatmap of the channel-wise L2 norm derived from the triplane representation across three orthogonal views.

#### 4.2.2 EMPIAR-10126

This dataset is composed of 45 247 particle images of the transient receptor potential melastatin (TRPM) ion channel, with a box size of 320×320 pixels and a pixel size of 1.046 Å. Figure 7A displays the reconstructions generated by cryoSPARC, cryoDRGN, cryoNeFEN, and cryoTriplane with C4 symmetry imposed. The results from Fourier-domain methods exhibit significant density fragmentation throughout the structure, indicating the relatively low SNR of this dataset. Consequently, this leads to structural information loss, as highlighted by the red dashed circles. In contrast, cryoTriplane successfully recovers these missing density features. Figure 7B presents the GS FSC curves and the local resolution distributions. Specifically, cryoTriplane achieves a 0.59 Å gain in global resolution (FSC_0.143_) relative to cryoNeFEN and significantly increases the proportion of high-resolution voxels (47.3% below 3 Å, compared to 21.8% for cryoNeFEN). Furthermore, as shown in Fig. 7C, cryoTriplane yields a higher sequence coverage of 62.6%, surpassing the 58.4% achieved by cryoNeFEN. These results further demonstrate the practical advantage of cryoTriplane in resolving fine structural details from challenging, low-SNR datasets. Figure 7D further comfirms that cryoTriplane successfully focuses its high-frequency encoding strictly on reconstructing intricate structural geometries.

**Figure 7:**
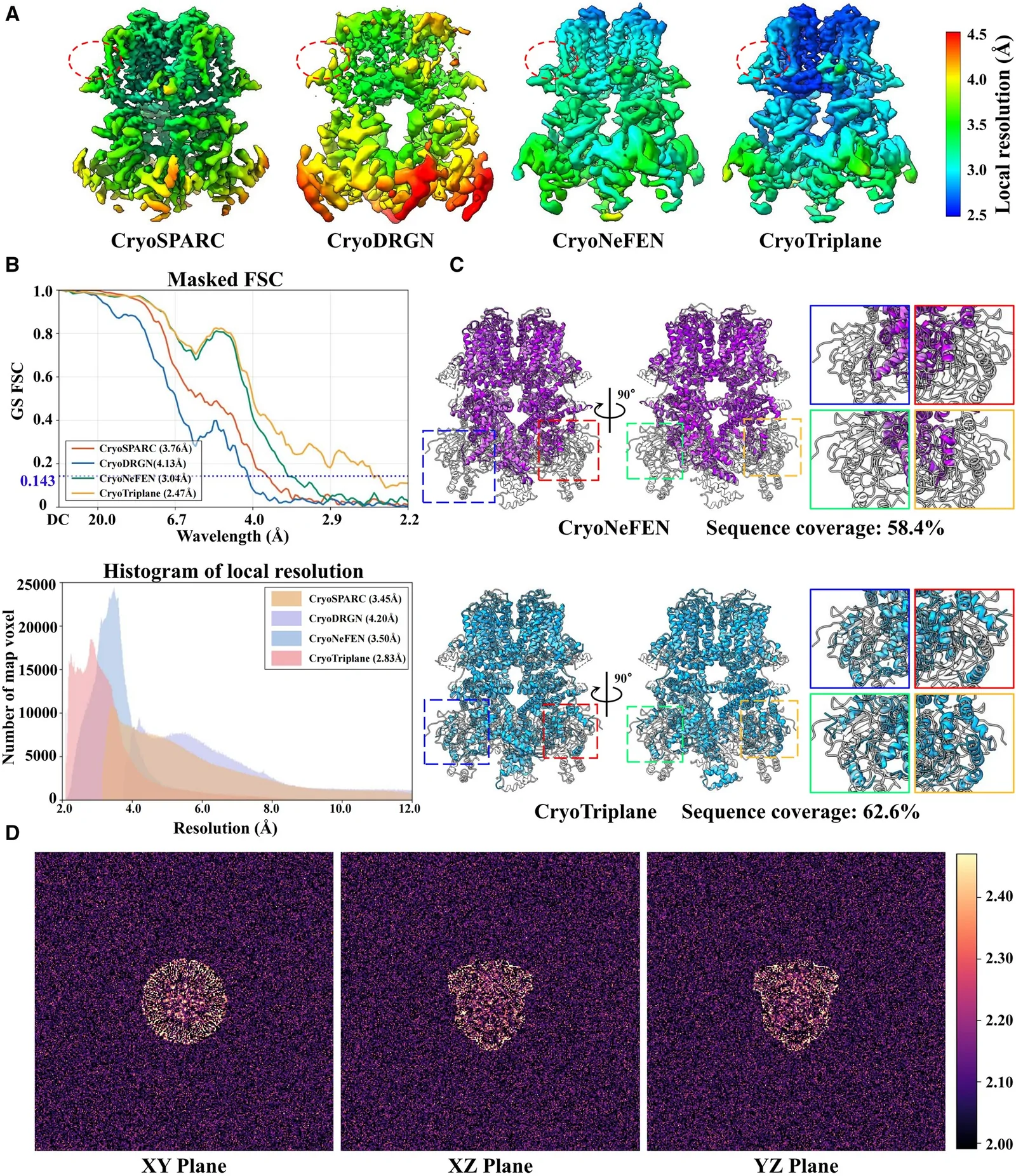
Results on TRPM4 ion channel (EMPIAR-10126). (A) Homogeneous reconstruction density maps generated by cryoSPARC, cryoDRGN, cryoNeFEN, and cryoTriplane under C4 symmetry. And each volume is colored according to local resolution to demonstrate the differences in reconstruction quality. The dashed circle shows the structural information loss phenomenon. (B) Plots of GS FSC curves and histograms of local resolution for maps reconstructed across different methods. (C) The atomic modeling results of cryoNeFEN and cryoTriplane under C4 symmetry, overlaid onto the reference PDB: 6BQR for comparison. The detailed views of the box region highlight the modeling difference. cryoTriplane improves the modeling sequence coverage by 4.2% compared to cryoNeFEN. (D) The heatmaps of the channel-wise L2 norm derived from the triplane representation across three orthogonal views.

#### 4.2.3 EMPIAR-10492

This dataset contains 29,183 particle images of severe acute respiratory syndrome coronavirus 2 (SARS-CoV-2) Spike glycoproteins, with an image size of 256×256 pixels and a pixel size of 1.098 Å. Figure 8A presents the density maps reconstructed by cryoSPARC, cryoDRGN, cryoNeFEN, and cryoTriplane with C3 symmetry imposed. Multiple views colored by local resolution collectively demonstrate the superior overall quality of cryoTriplane’s reconstructions. According to the quantified results shown in Fig. 8B, cryoTriplane achieves a 0.67 Å gain in global resolution (FSC_0.143_) relative to cryoNeFEN, with 69.6% of voxels resolving to better than 4 Å, a substantial improvement over cryoNeFEN’s 57.9%. This enhanced resolution significantly facilitates downstream *de novo* atomic modeling, as shown in Fig. 8C. CryoTriplane achieves a higher sequence coverage of 81.0%, compared to 75.5% for cryoNeFEN. Importantly, cryoTriplane’s superiority is most evident in the highly flexible apical region of the Spike protein. While traditional methods struggle to reconstruct this conformationally dynamic area, yielding poor-quality density, cryoTriplane successfully resolves its structural details, further validating the reliability and robustness of our framework. Figure 8D also confirms that cryoTriplane can achieve structure- aware encoding, thereby assisting the network in resolving complex structural details.

**Figure 8:**
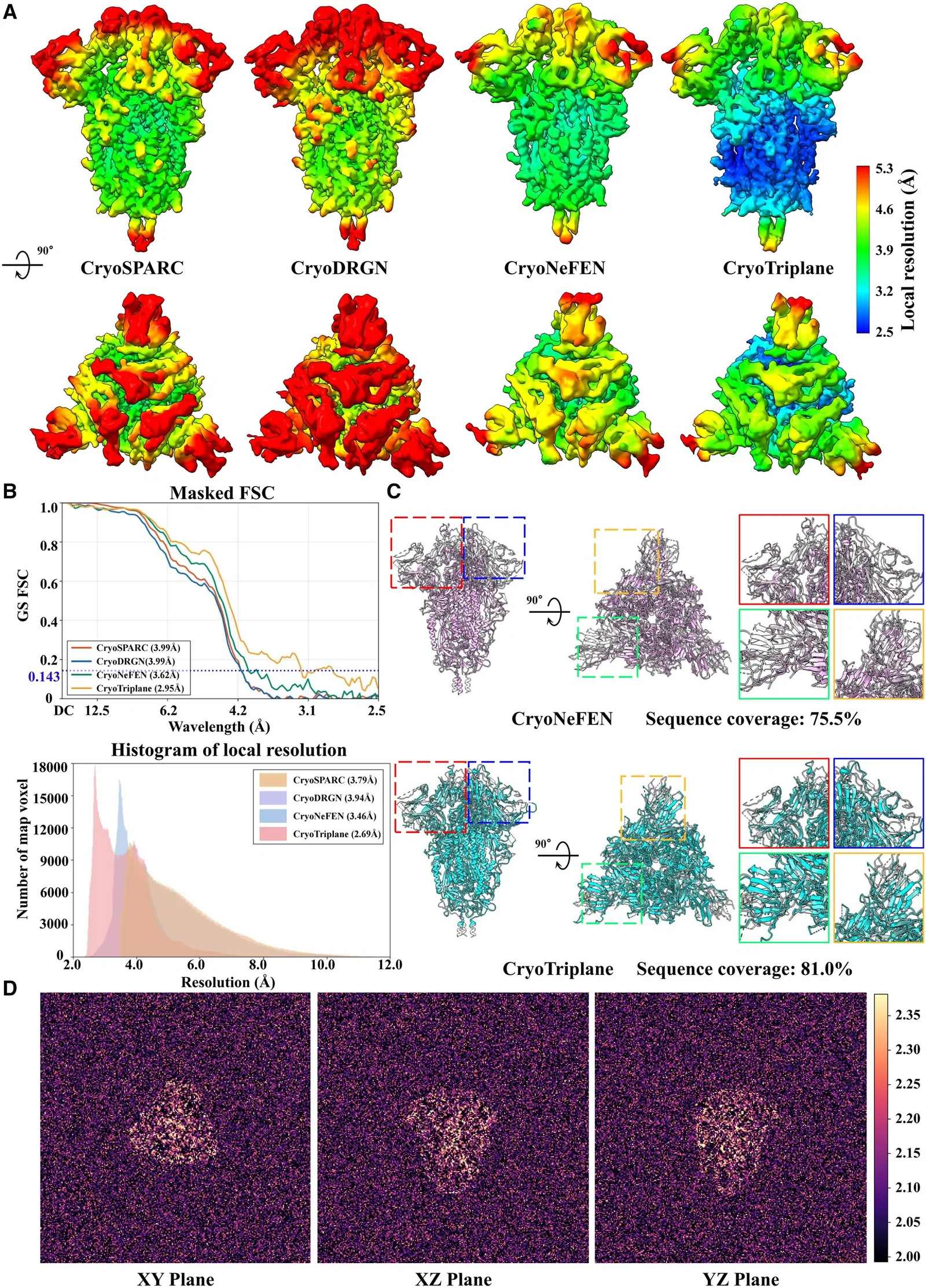
Results on SARS-CoV-2 Spike Protein (EMPIAR-10492). (A) Homogeneous reconstruction density maps generated by cryoSPARC, cryoDRGN, cryoNeFEN, and cryoTriplane under C3 symmetry. And each volume is colored according to local resolution to demonstrate the differences in reconstruction quality. (B) Plots of GS FSC curves and histograms of local resolution for maps reconstructed across different methods. (C) The atomic modeling results of cryoNeFEN and cryoTriplane under C3 symmetry, overlaid onto the reference PDB: 6ZWV for comparison. The detailed views of the box region highlight the modeling difference. CryoTriplane improves the modeling sequence coverage by 5.5% compared to cryoNeFEN. (D) The heatmaps of the channel-wise L2 norm derived from the triplane representation across three orthogonal views.

#### 4.2.4 EMPIAR-10786

There are 59 926 particle images of substance P-Neurokinin receptor G protein complexes, with an image size of 320×320 pixels and a pixel size of 0.835 Å. Figure 9A displays the homogeneous refined density from cryoSPARC alongside deep-learning-based reconstructed volumes from cryoDRGN, cryoNeFEN, and cryoTriplane, with all maps colored according to local resolution. As highlighted in the red and blue dashed circles, Fourier-based methods struggle to recover valid density for the head region of this protein complex, and produce noisy artifacts in the center area. The global and local GS FSC_0.143_ results in Fig. 9B reveals that cryoTriplane outperforms other methods in all evaluated aspects. For instance, cryoTriplane achieves a resolution better than 3 Å for 95.9% of its voxels, surpassing the 89.5% achieved by cryoNeFEN. Additionally, Fig. 9C illustrates the atomic model building results. As shown in the purple dashed circles at low contour level, cryoNeFEN’s reconstruction exhibits structural artifacts that result in erroneous local modeling. In contrast, the comparison in the blue dashed boxes underscores cryoTriplane’s superior capability in resolving local structural details. Figure 9D demonstrates that cryoTriplane adaptively allocates encoding capabilities to resolve high-resolution structural details.

**Figure 9:**
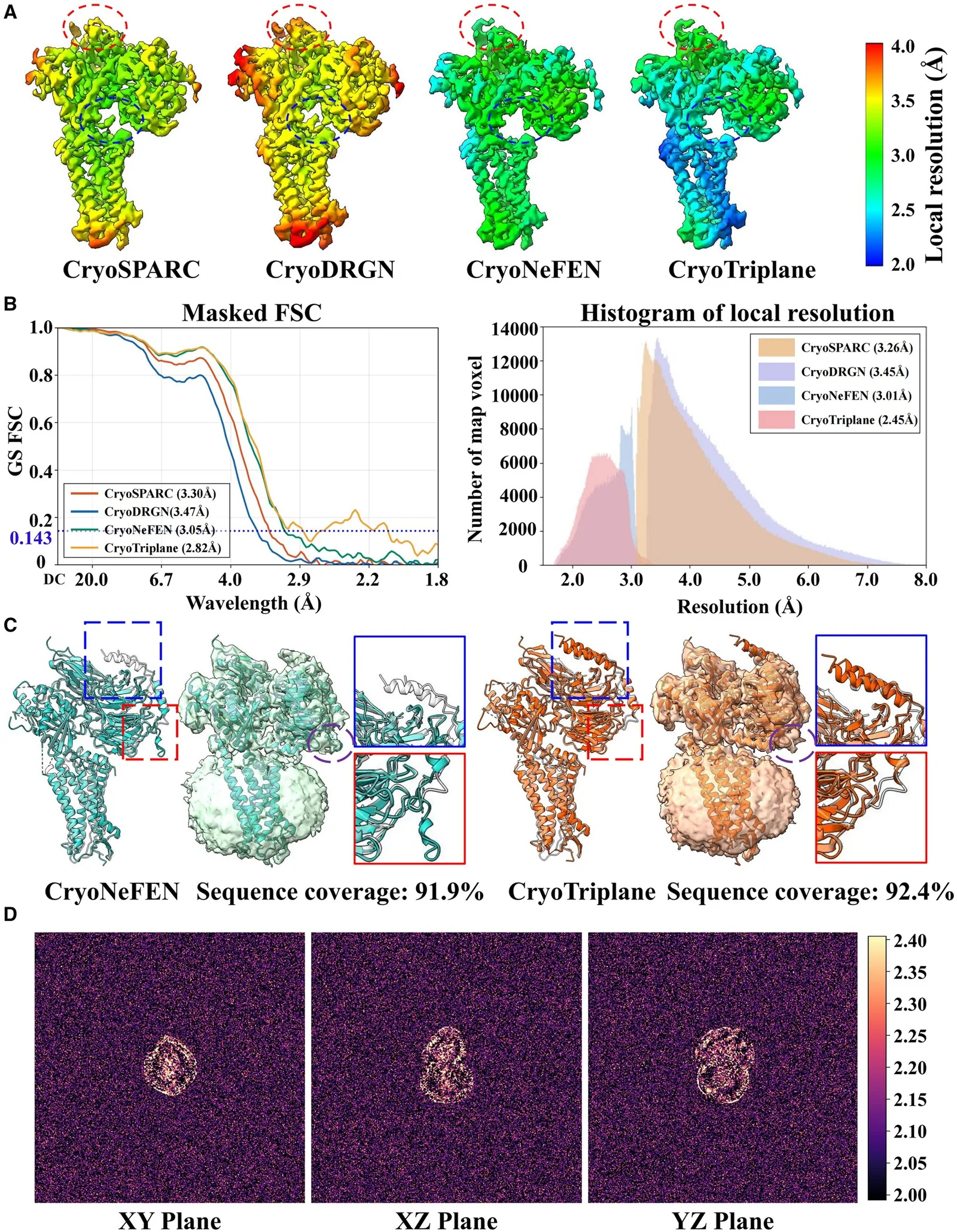
Results on substance P-Neurokinin receptor G protein complexes (EMPIAR-10786). (A) Homogeneous reconstruction density maps generated by cryoSPARC, cryoDRGN, cryoNeFEN, and cryoTriplane. And each volume is colored according to local resolution to demonstrate the differences in reconstruction quality. The dashed circles indicate structural loss (head) and artifacts (center), respectively. (B) Plots of GS FSC curves and histograms of local resolution for maps reconstructed across different methods. (C) The atomic modeling results of cryoNeFEN and cryoTriplane , overlaid onto the reference PDB: 7RMI for comparison. The detailed views of the boxed region highlight the modeling difference. The purple dashed circles indicate the density artifact that guides erroneous atomic modeling at the low contour level. CryoTriplane improves the modeling sequence coverage by 0.5% compared to cryoNeFEN. (D) The heatmaps of the channel-wise L2 norm derived from the triplane representation across three orthogonal views.

### 4.3 Comparisons on computational efficiency

To evaluate the practical feasibility of cryoTriplane, we analyze the trade-off between computational efficiency and reconstruction quality, comparing our method against the Fourier-domain baseline cryoDRGN and the real-space method cryoNeFEN, as summarized in Table 1. Because cryoNeFEN utilizes a fixed positional encoding with a frequency basis of L=32, it consumes less memory when decoding density at specific coordinates compared to cryoDRGN, which employs a larger frequency basis of L=D/2. However, because the number of active grid points in the real-space 3D volume remains significantly larger than that of the 2D Fourier slices even after mask filtering, training and rendering projection images in the real-space domain still demand more memory and computational time. Building upon the real-space framework, cryoTriplane introduces three orthogonal feature planes whose resolution scales with the image size, which inevitably increases both memory consumption and training time compared to cryoNeFEN. Nonetheless, this computational overhead is fully justified by the superior reconstruction resolution achieved by cryoTriplane.

**Table 1:** Comparison of computational cost and reconstruction performance across four real-world datasets.

		EMPIAR-10005 (*D* = 256, *N* = 35 645)	EMPIAR-10126 (*D* = 320, *N* = 45 247)	EMPIAR-10492 (*D* = 256, *N* = 29 183)	EMPIAR-10786 (*D* = 320, *N* = 59 926)
CryoDRGN	Params.	∼3.94M	∼4.13M	∼3.94M	∼4.13M
	Train time(min/epoch)	∼1.77	∼4.00	∼1.43	∼5.17
	Resolution	3.66 Å	4.13 Å	3.99 Å	3.47 Å
CryoNeFEN	Params.	∼0.18M	∼0.18M	∼0.18M	∼0.18M
	Train time(min/epoch)	∼10.05	∼30.50	∼13.12	∼25.83
	Resolution	3.02 Å	3.04 Å	3.62 Å	3.05 Å
CryoTriplane	Params.	∼3.33M	∼5.10M	∼3.33M	∼5.10M
	Train time(min/epoch)	∼11.17	∼38.58	∼18.30	∼28.50
	Resolution	2.43 Å	2.47 Å	2.95 Å	2.82 Å

### 4.4 Ablation studies on model design

The main novelty of cryoTriplane compared to similar deep learning methods lies in its hybrid positional encoding strategy. To validate the effectiveness of the proposed components and regularization in cryoTriplane, we conduct comprehensive ablation studies on EMPIAR-10005. Specifically, we compare cryoTriplane with five distinct configurations: (i) the baseline model cryoNeFEN, which uses only the fixed positional encoding; (ii) cryoTriplane[no fixed PE], which employs only the triplane-based positional encoding; (iii) cryoTriplane[sum] and (iv) cryoTriplane[concat], where the original element-wise multiplication is replaced by summation and concatenation, respectively; and (v) cryoTriplane[no TV], which is trained without TV regularization. The quantitative results and visual comparisons are presented in Fig. 10.

**Figure 10:**
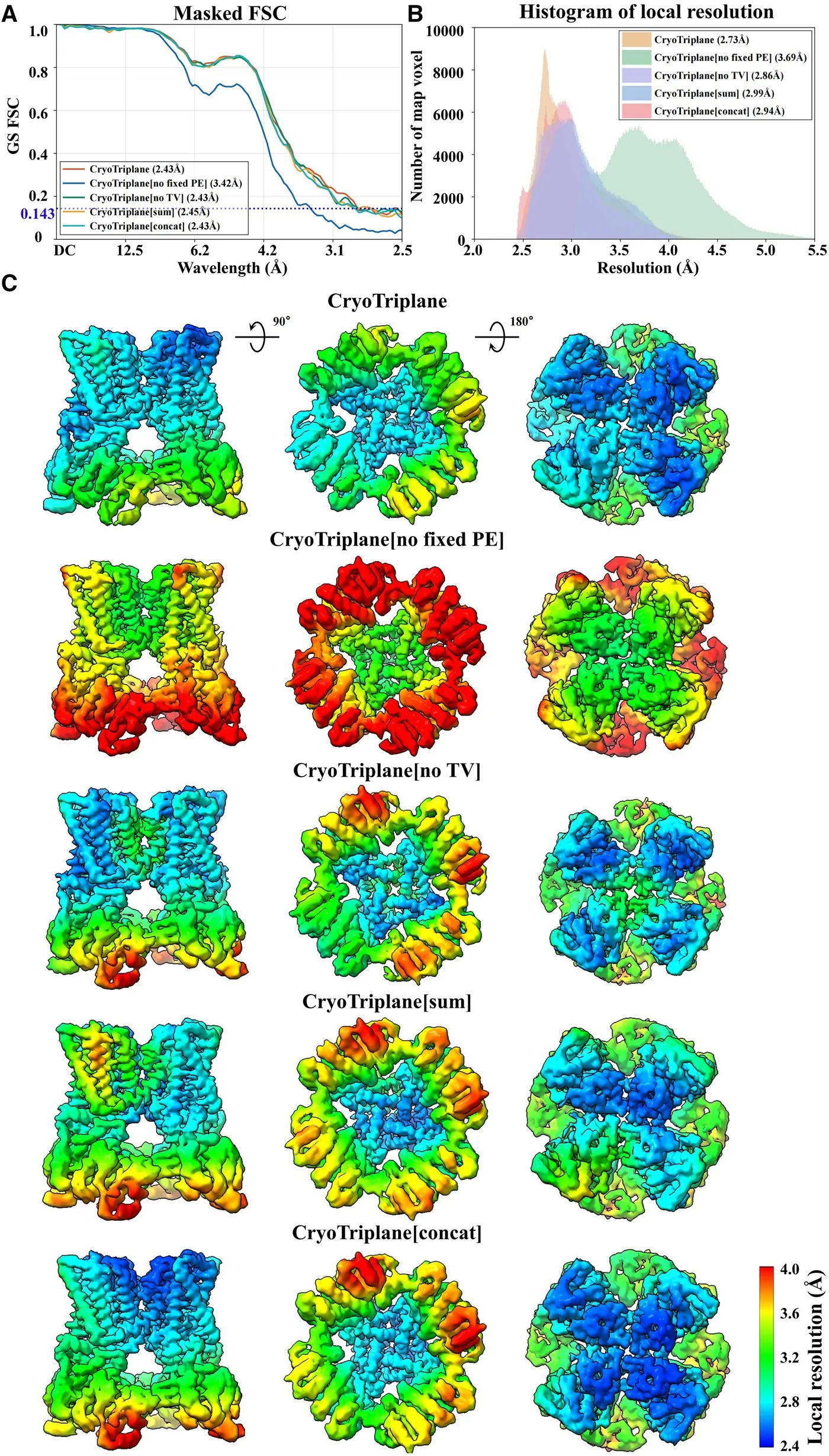
Ablation results on hybrid positional encoding strategy. (A) GS FSC curves for cryoTriplane with various ablation configurations. (B) Corresponding histogram of local resolution. (C) Homogeneous reconstruction density maps colored by local resolution for different variants under C4 symmetry.

First, we analyze the joint contribution of fixed positional encoding and triplane features. Experimental results show that neither adopting the fixed positional encoding alone (Fig. 6) nor utilizing the triplane representation alone achieves the optimal reconstruction resolution. This performance gap highlights a strong complementarity between these two representations. The fixed positional encoding provides global spatial continuity, which prevents grid-like discretization artifacts and stabilizes low-frequency reconstruction under low SNR conditions. Meanwhile, the triplane-based representation dynamically stores spatially localized, adaptive high-frequency features. Integrating both representations allows cryoTriplane to leverage continuous global mapping while preserving high-resolution local details. Furthermore, omitting the TV regularization term leads to a noticeable degradation in local resolution. Since cryo-EM images typically suffer from extremely low SNR, the TV loss acts as an effective edge-preserving spatial smoothness prior, suppressing high-frequency background noise in the reconstructed volume without blurring sharp macromolecular boundaries.

Second, we evaluate the impact of different feature fusion strategies. CryoTriplane employing multiplication-based aggregation reaches a peak resolution of 2.73 Å, significantly outperforming the variants utilizing summation- or concatenation-based aggregation, both of which only reach approximately 3.0 Å. This difference arises from how features are coupled across dimensions. Element-wise multiplication enforces a joint spatial constraint, restricting feature activation to the co-localized intersection of the three orthogonal planes. In contrast, summation independently overlays plane features, allowing signals to propagate along the projection axes without mutual constraint. Concatenation merely increases feature dimensionality without explicit plane interaction, relying entirely on the subsequent network layers to learn spatial correlations implicitly. This increases optimization complexity and results in suboptimal convergence. The reconstructed density maps colored by local resolution (Fig. 10C) visually confirm that the multiplication-based triplane aggregation strategy achieves the best reconstruction quality.

## 5 Conclusion

In this paper, we present cryoTriplane, a novel high-resolution 3D homogeneous reconstruction framework for cryo-EM SPA datasets, which demonstrably surpasses state-of-the-art methods. While recent real-space approaches mitigate issues common to Fourier-based methods, their performance remains fundamentally constrained by the inherent spectral bias of MLPs, which limits their capacity to represent high-frequency structural details. cryoTriplane aims to alleviate this bottleneck by introducing a structure-aware representation strategy by combining triplane-based explicit feature representation with fixed positional encoding. This approach enables the network to adaptively and uniformly learn features across all spatial frequencies, providing a structure-aware and adaptive basis that effectively captures high-frequency information. As a result, cryoTriplane achieves significant improvements in both global and local resolution, which directly translates to more complete and accurate atomic models, thereby advancing the reliability of downstream structural analysis.

## Data Availability

The data underlying this article are available in EMPIAR(https://www.ebi.ac.uk/empiar), under accession code EMPIAR-10005, EMPIAR-10126, EMPIAR-10492,EMPIAR-10786.
